# Experiment on Compressive Properties and Microscopic Analysis of Sea Sand Geopolymer-Based Recycled Concrete

**DOI:** 10.3390/ma17010028

**Published:** 2023-12-20

**Authors:** Deyi Xu, Guanfeng An, Yuliang Chen, Zhihua Liu, Xiangli Liu

**Affiliations:** 1 Guangzhou Municipal Construction Co., Guangzhou 510062, China; xudeyi195@163.com (D.X.);; 2 School of Civil Engineering, Guangxi University of Science and Technology, Liuzhou 545006, China; zhualiu258@163.com (Z.L.); liuxiangli991120@163.com (X.L.); 3 Key Laboratory of Disaster Prevention & Mitigation and Prestress Technology of Guangxi Colleges and Universities, Liuzhou 545006, China

**Keywords:** geopolymer material, recycled concrete, sea sand, curing age, compression constitutive model

## Abstract

For marine and coastal engineering, construction resources have become scarce due to a limited local supply. Sea sand geopolymer-based recycled concrete (SSGRC) is an innovative cementitious material known for its eco-friendly benefits and corrosion resistance. This study explores the mechanical properties of SSGRC. The influences of the replacement rate of mineral slag, alkali activator concentrations, fine aggregate types, and curing ages on the compression strength of SSGRC were studied. The failure mechanism was analyzed using the failure patterns and compressive stress–strain curves. The results show that sea sand had a positive effect on geopolymer-based material. The SSGRC reached peak strength with an alkali activator concentration of 10 mol/L and a mineral slag replacement rate of 60%. The maximum stress and strain increased with an increasing curing age. The ratios of strength to the peak value were 55% and 85% after 1 day and 7 days, respectively. Using SEM, in the last hydration stage, the C-(A)-S-H gel was formed with a dense microstructure, and the geopolymer exhibited a favorable bonding performance. The constitutive models describing the complete stress–strain relationship under compression were developed.

## 1. Introduction

With the rapid development of the emerging marine industry and seaport infrastructure in island and coastal regions, there is a huge demand for construction materials. In marine engineering, structures are susceptible to severe corrosion due to the corrosive environment. Hence, concrete is regarded as the most basic material used for marine and port engineering around the world [1]. However, the tremendous consumption of raw materials for use in concrete not only leads to a decrease in river sand and coarse aggregates but also produces high levels of carbon dioxide (CO_2_) emissions [2]. With the high demand for construction materials, over-extraction has a harmful impact on ecosystems and river resources [3]. In addition, the cost of construction in coastal areas increases significantly due to the expenses for transporting the major materials, as these areas heavily rely upon the inland areas. Notably, the concrete waste from island and coastal areas is expensive to dispose of because it must be transported long distances. To solve these problems, it is imperative to explore the creation of new construction materials that are more environmentally friendly yet remain easily accessible in order to partially or completely replace the aggregate resource.

Concerning the shortage of raw construction materials in marine and coastal regions, sea sand recycled coarse aggregates are regarded as a promising solution [4]. Due to defects caused by microcracks in the interface transition zone (ITZ) of recycled coarse aggregates (RCAs), a deterioration in the mechanical performance of recycled aggregate concrete (RAC) was observed [5]. Hence, various methods have been proposed to improve the properties of RAC, such as adding fibers to RAC and removing the older mortar on its surface [6,7]. Sea sand exhibits low levels of clay and the desired size compared to manufactured sand and river sand. Due to the abundance of sea sand resources, the utilization of sea sand as a fine aggregate in RAC is also conducive to the creation of sustainable concrete materials. Therefore, many studies have focused on the detailed behavior of sea sand recycled aggregate concrete. Huang et al. [8] investigated the effect of sea sand and RCA content on the properties of concrete. It was concluded that the compressive strength increases with a decrease in the RCA replacement ratio and an increase in sea sand content. Feng et al. [9] reported that an increase of approximately 20% was observed in the compressive strength at 28 days for RAC containing sea sand and seawater compared to RAC with the same water-to-cement ratio. Zhang et al. [10] applied the seawater–sea sand recycled coarse aggregate concrete using a scale column. They found that SSRCA columns outperform RAC columns in terms of strength and deformability, with a peak load capacity that is approximately 17% higher. The applicability of the existing stress–strain relationship model for analyzing SSRAC was evaluated in a study by Xiao et al. [11]. Based on the above research, they concluded that the reduction in compressive strength caused by a defect in the RCAs would be offset by the seawater and sea sand. However, the high levels of sulfate ions and chloride ions in the sea sand accelerated the production of gypsum and ettringite, which led to a matrix crack and increased the risk of an alkali–aggregate reaction (AAR) [12,13]. Therefore, the cleaning and chemical treatment of sea sand are two necessary steps. Due to the impurities in the recycled aggregates, they are vulnerable to the strong acid from the chemical treatment. Moreover, the hydration products of Portland cement (PC) are dominated by C-S-H gel and Ca(OH)_2_, making this type of cement vulnerable to acid and posing a risk to the marine environment.

Geopolymers, a new silicon–aluminum cementitious material, have attracted considerable attention in the area of green concrete. The production of CO_2_ generated from geopolymeric cement was 1/6 times less than that produced by Portland cement per ton [14]. Geopolymers can be obtained from waste materials or by-products such as fly ash, which is regarded as an eco-friendly and cost-effective material [15]. The geopolymerization process includes the formation of aluminosilicate within a three-dimensional network due to the reaction of materials containing alumina and silica with alkaline liquids [16]. When it makes contact with the alkaline activating solution, the solid aluminosilicate dissolves into aluminate and silicate species. Then, the monomers combine into dimers, trimers, and tetramers. When the solution reaches saturation, the aluminosilicate gel is formed and precipitated. A new gel is formed due to the increase in the amount of silicon dissolved in the solution, and the silicon enriches the aluminosilicate gel [17,18]. In addition, the strong binding property and low porosity of various geopolymer mixtures make geopolymers ideal materials for use in marine environments. The hydration products, including calcium aluminosilicate hydrates (C-A-S-H) and sodium aluminosilicate hydrates (N-A-S-H) in slag–metakaolin (SGMK) and slag–fly ash (SG-FA), for instance, exhibit an outstanding ability to resist chemical attacks [19]. Zhang et al. [20] reported that an appropriate slag and fly ash ratio could improve the chloride binding capacity due to the physical adsorption of hydrate products such as N-A-S-H, which could be a promising application under marine conditions. For this reason, incorporating sea sand into geopolymer concrete is another potential method that can be carried out. Wang et al. [21] applied the geopolymer as a binding matrix in coral aggregate concrete, and the concrete achieved a compressive strength of up to 60 MPa using reasonable methods. Das et al. [22] presented an optimized utilization process using Na_2_O% with alkali proportions to optimize the compressive strength of geopolymer concrete that contains waste concrete. Nguyen et al. [23] found that the optimal value of the alkaline-to-fly ash ratio is 0.35–0.45 for sea-sand-based geopolymer concrete, which is similar to that for river-sand-based geopolymer concrete. Shi [24] studied the compressive properties of geopolymer recycled concrete with a coarse aggregate type as the design parameters. It was found that the compressive strength of geopolymer recycled concrete was effectively improved, as it could be equivalent to or even higher than that of ordinary concrete. Salim et al. [25] concluded that geopolymers and mineral admixtures efficiently mitigate the AAR if they are used at an appropriate percentage. By contrast, Shinde et al. [26] found that a reduction from 13.07% to 17.62% in the compressive strength of untreated sea sand geopolymer concrete was observed compared to that using river sand. Rashad et al. [27] reported that the reduction in the compressive strength of geopolymer concrete decreased with the increasing content of sea sand. Due to the importance of constitutive behavior, many research studies have focused on establishing an accurate model to predict the behavior of reinforced geopolymer structures. In comparison with the ACI model, Diaz et al. [28] put forward equations to determine the flexural strength and static elastic modulus. Noushini et al. [29] developed a method to determine the complete stress–strain behavior under uniaxial compression of fly ash-based geopolymer concrete. Zhang et al. [30] put forward two empirical equations to predict the residual compressive strength of geopolymer concrete after exposure to different temperatures.

As the studies above demonstrate, there are few articles that focus on the mechanical properties of geopolymer concrete that incorporate recycled concrete sea sand as a fine aggregate and recycled concrete as a coarse aggregate. Based on the finding of the positive effect of sea sand on RAC and the contradictory results regarding the effect of sea sand on geopolymer concrete, the performance of SSGRC has not yet been clearly determined. To this end, this study investigated the effect of the slag replacement ratio, NaOH solution concentration, curing age, and the ratio of sea sand as a fine aggregate to recycled concrete as a coarse aggregate on the mechanical properties of geopolymer recycled concrete. Also, Scanning Electron Microscope imaging was carried out to examine the SSGRC. Furthermore, the compressive constitutive model of SSGRC was proposed and evaluated.

## 2. Experimental Results

### 2.1. Experimental Materials

**Figure 1:**
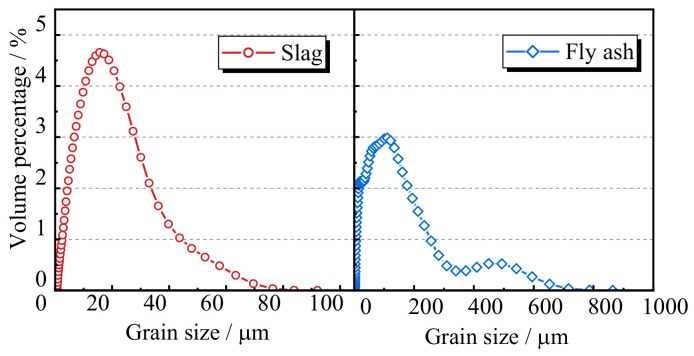
The grading curve of the slag and fly ash.

**Figure 2:**
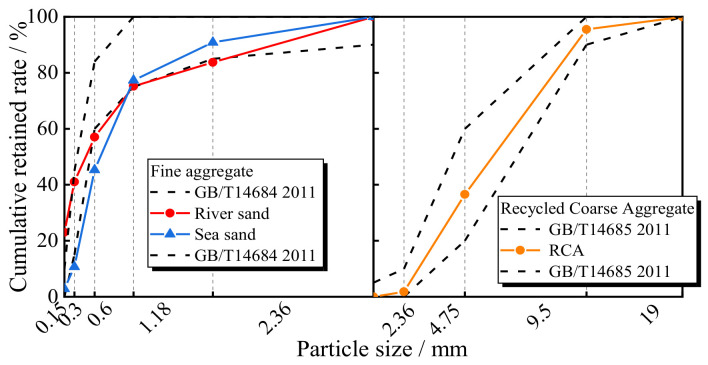
The grading curve of the aggregates.

The fly ash and slag were obtained as the by-products of coal combustion and the smelting of metallic ore, respectively, which were carried out in Henan Province, and these materials were used to replace cement as the inorganic bond material. The median particle size of grade II fly ash used in this study was 83 mm, while the grade S95 slag had a median particle size of 16 mm. The chemical compositions of the fly ash and slag are shown in Table 1, and the volume percentage curves are shown in Figure 1. The original RCAs were produced after crushing and sieving the waste concrete using an experimental beam in Guangxi Province. Their particle sizes were within 5–20 mm, and the grading curve is plotted in Figure 2. According to standard GB/T 25177-2010 [31], the basic properties of the coarse aggregates are shown in Table 2. The main constituents of the fly ash, such as SiO_2_, Al_2_O_3_, and CaO, make up the primary chemical composition for the polymerization process, leading to a 3D tetrahedron structure and the formation of the Si-O-Al-O bond. Moreover, the Fe_2_O_3_ and CaO in the fly ash can produce good alkaline activity for the polymerization process. The moisture content of the sea sand from the South China Sea was 5.82% and its fineness modulus was *M*_x_ = 2.8, which indicates that it is a medium-grade sand. River sand with medium grading, a moisture content of 1.99%, and a fineness modulus *M*_x_ of 2.97 was prepared and obtained in Guangxi Province. Except for chemical salt and sea shells, there were slight variations between the river sand and sea sand. The artificial alkali-activated solution from Hunan Province was prepared by mixing sodium silicate and caustic soda in a ratio of 2 by mass. The purity of the flaked sodium hydroxide was 98%.

### 2.2. Specimen Design

In this work, the replacement ratio of slag as a substitute for fly ash, the NaOH solution concentration, the type of fine aggregate, and the curing age were used as the influencing factors for the specimen design. Five curing ages, including 1 day, 3 days, 7 days, 14 days, and 28 days, were chosen to observe the trend in mechanical properties with increasing time. “D” stands for curing day. Three componential effects were discussed. First, the different activator (NaOH solution concentration) effects, selected as 8%, 10%, and 12% (by weight), were adopted, while the second number on the label denoted the NaOH solution concentration. Second, the slag replacement ratios, including 20%, 40%, and 60% (by weight), were analyzed within the design mix. The slag replacement ratio was expressed as the third number in the specimen label. Third, two mixes of fine aggregates were studied. One was entirely river sand and the other was half river sand and half sea sand.

The 42 cubic specimens with dimensions of 150 mm × 150 mm × 150 mm were designed, and all of the mix proportions are listed in Table 3. Taking “D1-10-60-S” as an example, D1 denotes a curing age of 1 day; “-10” and “-60” represent the NaOH solution concentration and slag replacement rate, respectively; and S denotes sea sand.

### 2.3. Test Method

To evaluate the mechanical properties of specimens, two kinds of analyses were used: a compressive strength test and an SEM test. For the compressive strength test, the MATEST 5000 kN testing machine, developed in Italy and controlled by a computer, was used in this study. The loading system adopted the method of displacement control. In the displacement-controlled stage, the loading rate of 1.2 mm/min was applied to impose the vertical load until the specimen was damaged or the axial deformation was too large. The data acquisition system continuously collected the load and displacement data in real time. SEM analysis was used to define the microstructure, particles, and formation of specimens. SEM analysis was conducted on each of the three concretes from every group after the compression test. One from each group was selected to demonstrate the significant morphological characteristics, which include the presence of voids around aggregates, the presence of microcracks, the intermediate product of the geopolymerization process, and so on. The pixel resolution of the samples ranged from 20 μm to 300 μm.

## 3. Test Results and Failure Mechanism Analysis

### 3.1. The Compressive Failure Pattern

**Figure 3:**
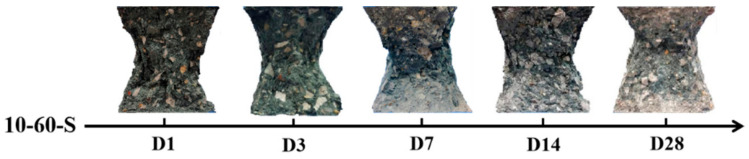
The failure patterns at different curing ages.

Figure 3 shows the failure pattern of geopolymer concrete with sea sand at different curing ages. The specimens were selected using the same design mix that was fixed with a NaOH solution concentration of 10 mol/L and a slag replacement rate of 60%. The failure process was similar to that of ordinary concrete. Firstly, vertical cracks gradually formed on the surface. Then, minor cracks gradually led to cracks throughout the entire specimen. In the post-peak stage, spallation on the lateral sides was observed. As shown in Figure 3, through the comparison of the failure patterns at different curing ages, it can be found that with the increase in curing age, the failure surface color of the specimens changes from black to light gray. Generally, during the destruction process, the spalling of aggregates from the geopolymer cement is accompanied by a “squeak” sound. With the increase in curing age, the span of the “squeak” sound increased. This phenomenon illustrates that the fly ash and slag activated by the alkali-activated solution are greatly affected by the curing time.

**Figure 4:**
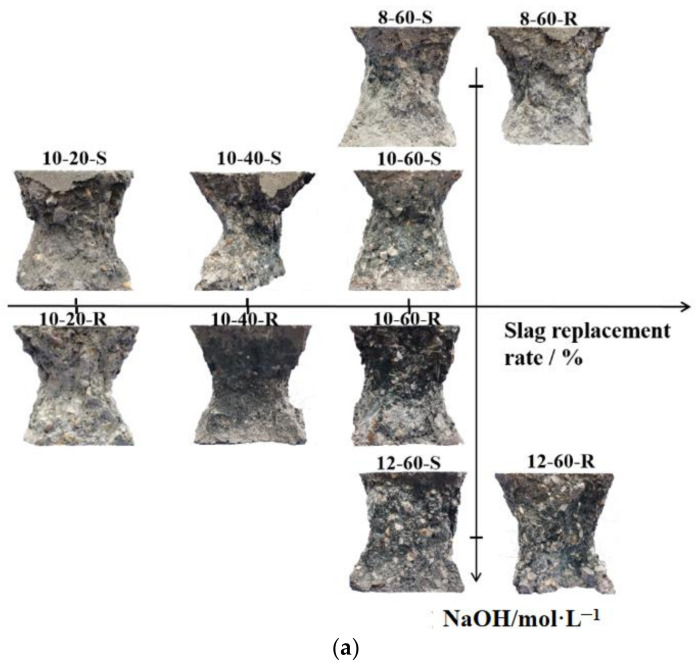

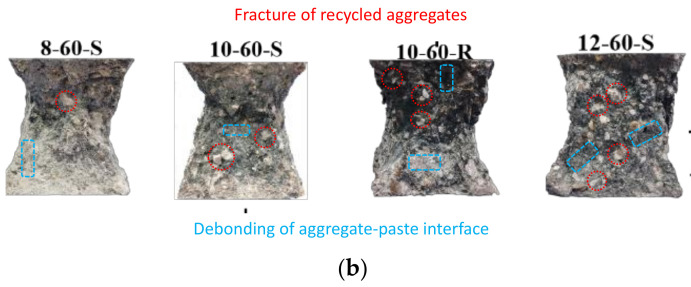
The failure patterns with the different slag replacement rates and alkali activator concentrations after 28 curing days. (**a**) The failure patter with different slage replacement rate and NaOH molarity; (**b**) The failure surface.

The failure patterns of specimens with different slag replacement ratios and alkali activator concentrations are shown in Figure 4. The horizontal axis in Figure 4a denotes the variation in the slag replacement ratio, while the vertical axis represents the variation in the NaOH solution concentration. As displayed in Figure 4b, with the increasing molarity of the NaOH solution and the slag replacement rate, more broken recycled coarse aggregates, represented by the white color, are observed on the failure surface. Moreover, the increasing molarity of the NaOH solution results in the failure surface turning from light gray to black. It can be observed that with the increase in the NaOH solution molarity, the failure pattern demonstrates more RCAs being fractured rather than interface debonding between the matrix and aggregates. The increasing molarity of the NaOH solution accelerates the dissolution of silicon and aluminum ions from the slag and fly ash. Simultaneously, this leads to the increase in Na+ ions in the solution, which is the key element for the geopolymerization process. The sufficient number of ions is helpful in combining it with the N-A-S-H gel product, contributing to the material’s early strength. This can explain how the NaOH solution concentration helps improve the bonding strength of the geopolymer binders. Through the vertical axis, it can be observed that the color of the failure surface turns dark with the increase in NaOH solution concentration. Alkali-activated concrete commonly exhibits a dark color. A certain amount of white unactivated powder is detected on the fractured surface of the 8-60-S specimen with an 8 mol/L NaOH solution concentration. This can be attributed to the fact that a high NaOH solution concentration can accelerate the speed of the activation process of fly ash and slag.

From the horizontal axis, with the increase in the slag replacement ratio, less powder is observed on the destruction interface. It can be concluded that a higher slag replacement ratio can result in a more complete activation reaction and better bonding performance between aggregates and the cementitious matrix.

### 3.2. Micromorphology Analysis

**Figure 5:**
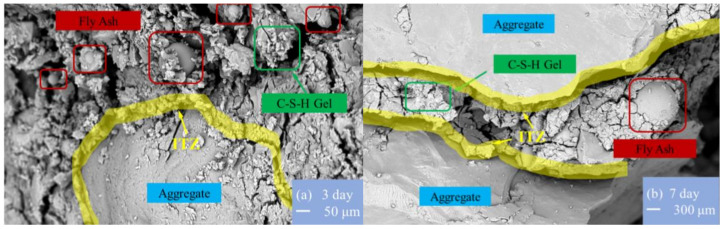

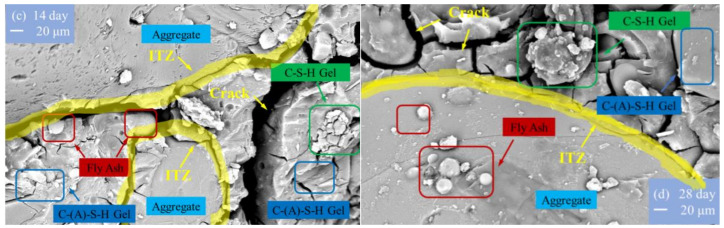
SEM images of SSGRC.

The Scanning Electron Microscope (SEM) was also used to analyze the microstructure. Figure 5 presents the results of the microstructural observation of the geopolymer concrete with sea sand at different curing ages. As shown in Figure 5a, for the specimen with a curing age of 3 days, there are some partially reacted and unreacted fly ash particles remaining on the surface, while the C-S-H gel, regarded as an unstable microstructure, is visible on a significant proportion of the surface. Utilizing the SEM also revealed a small portion of C-(A)-S-H gel acting as a stable structural product, which was formed by the chemical reaction between the fly ash, slag, and C-S-H gel. There were numerous discrete microcracks observed in the C-S-H gel paste, resulting in poor bonding behavior. These reasons explain the reduction in compressive strength for the sea sand geopolymer concrete at an early stage. As shown in Figure 5b–d, since the fly ash particles were rarely observed, the alkaline activation reaction was almost completed. Subsequently, the free Al(OH)_4_^−^ group in the polymer’s internal system reacts with the C-S-H gel to produce a stable C-(A)-S-H gel microstructure in the later hydration stage. The final product exhibits a dense structure, and this explains why the compressive strength of the SSGRC increases as the curing age increases. As shown in Figure 5, compared to the specimen after 7 days, 14 days, and 28 days, the specimen at the curing age of 3 days has more visible microcracks and fly ash particles scattered across the concrete. This means that the geopolymerization reaction is still continuing, and the fly ash exhibits a spherical particle form. As the number of curing days increases, a reduction in fly ash is observed and more C-(A)-S-H gel filling the microcracks is produced, resulting in a dense microstructure. For the curing ages of 14 and 28 days, the hydration process is more mature and the sample appears free of voids. The main cracks along the interface transition zone (ITZ) of the aggregate for the specimen after 7 days of curing are more apparent than those after 14 and 28 days of curing. The specimen after 28 days of curing shows almost no unreacted fly ash, and cracks are mainly observed in the geopolymer paste instead of along the ITZ. The geopolymer exhibits a favorable bonding performance between the paste and the RCAs.

### 3.3. The Curves of Compressive Stress–Strain for the Specimens

The compressive strengths of the specimens are shown in Table 4. All of the compressive strengths are calculated according to GB50107-2010 [32].

**Figure 6:**
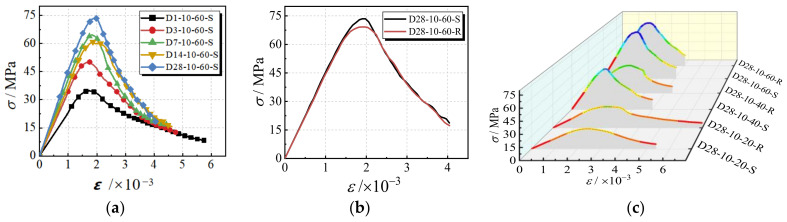
The curves of compressive stress–strain. (**a**) Different curing ages; (**b**) Different fine aggregates; (**c**) Different slag replacement rates.

The compressive stress–strain curves for the specimens are illustrated in Figure 6. It can be observed in Figure 6a that the slopes (modulus of elasticity) and the peak secant modulus at the ascending stage gradually improve with the increasing curing age. Additionally, after 7 days of curing, the increment rate of the modulus of elasticity slows down. The peak strain increases slightly with the increase in the curing age. At the descending stage, the curves for the specimen with longer curing ages descend more sharply than that with a 1-day curing age. The specimens with a long curing age show brittle failure patterns.

The effect of different fine aggregate types on the compressive behavior is shown in Figure 6b. Except for the peak stress, the behavior of the specimens with different fine aggregate types is highly similar. This can be attributed to the same failure pattern resulting from the voids in the geopolymer paste.

The relation curves of compressive stress–strain with different slag amounts and fine aggregate types are shown in Figure 6c. The compressive strength increases significantly with an increase in the slag replacement ratio because the higher geopolymerization of Si-O-Si or Si-O-Al is derived from the alkali-activated reaction of the slag. It can be seen that the elastic modulus shows a tendency to increase with the increase in the slag replacement ratio. This can be explained by the different reaction products. Due to the inclusion of slag, there is more C-A-S-H gel formed during the geopolymerization process, while the main reaction product in the geopolymeric concrete without slag is N-A-S-H gel. Additionally, Young’s modulus of the C-A-S-H gel is higher than that of the N-A-S-H gel. In conclusion, the main load is represented by the recycled coarse aggregate and cement paste in the ascending segment of the curve that corresponds to the initial loading process. After reaching the peak stress, with the development of cracks, the hole defects and voids are enlarged and the curve decreases rapidly in the descending segment.

## 4. Analysis of Influencing Factors

### 4.1. The Effect of Curing Ages on Compressive Strength

**Figure 7:**
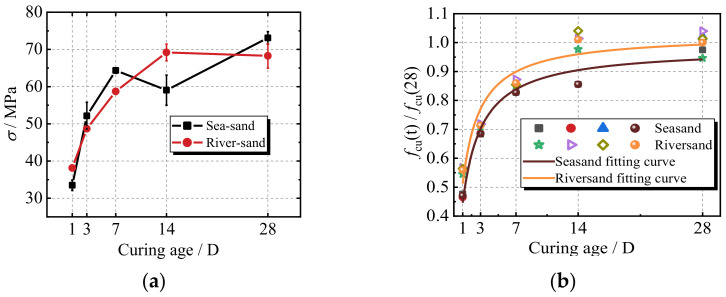
The effect of different curing ages on the compressive strength. (**a**) Different curing ages; (**b**) The relationships between the normalized compressive strength and curing age.

Figure 7a shows the relationship between the cube compressive strength and curing age for geopolymer concrete with sea sand and river sand. With an increase in curing age, the compressive strength of the specimens with the two series of fine aggregates increases. In the first 7 days, a rapid increase is observed in the compressive strength. After 7 days, the strength of both the specimens with sea sand and river sand develops slowly. During the stage of rapid growth in strength, the strength of the specimen with sea sand is comparable to that of the specimen with river sand but is even higher. The compressive strength of geopolymer concrete at 1 and 7 days can reach 56% and 86%, respectively, of that of the 28-day compressive strength. It should be noted that geopolymer concrete exhibits a rapid increase in compressive strength. For the 28-day strength, the sea sand-based geopolymer sample is about 7% higher than that of the river sand sample. This can be explained by the fact that, compared with river sand, sea sand can supply enough silicon dioxide, calcium, and magnesium, which are the key raw materials needed to produce more silicate and aluminosilicate within the geopolymer reaction process. As a result, a dense network structure is established and the strength increases.

Based on a previous study [33], the regression analysis was carried out, and as shown in Equations (1) and (2), the predicted models for the cube compressive strength of the geopolymer concrete were proposed. In Equations (1) and (2), *t* represents the curing age. The predicted versus experimental values for the newly developed models of the relationship between the normalized compressive strength and curing age are plotted and shown in Figure 7b. In both of the two models, the predicted and measured values fall on and are evenly scattered around the line of equality, indicating that the models are fairly accurate in their predictions. The data predicted by the proposed two models are in agreement with the experimental results, with an error band of less than 10%.

(1) $$f_{\mathrm{cu}}(t^{S})=f_{\mathrm{cu}}(28)\times \left(\frac{t}{1.224+1.018t}\right),1d\leq t\leq 28d,(R^{2}=0.944)$$

(2) $$f_{\mathrm{cu}}(t^{R})=f_{\mathrm{cu}}(28)\times \left(\frac{t}{0.980+0.972t}\right),1d\leq t\leq 28d,(R^{2}=0.915)$$

### 4.2. The Effect of Slag Rate Concentration and NaOH Molarity on Compressive Strength

**Figure 8:**
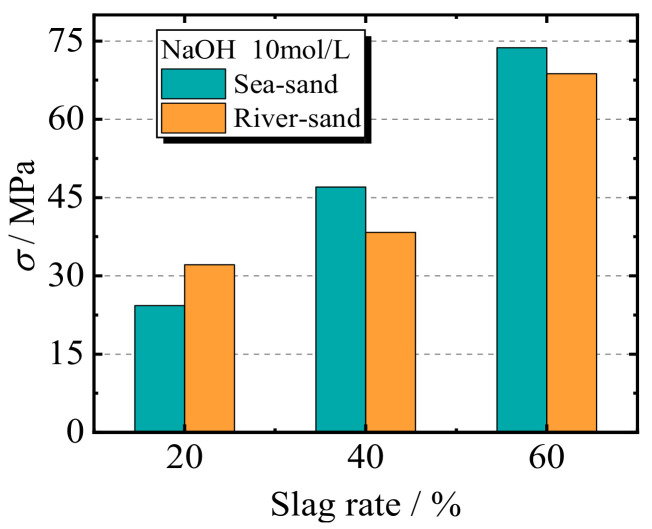
The effect of slag replacement rates.

Figure 8 shows the comparison between the peak strengths of specimens with different slag replacement rates at the same curing age of 28 days, while the NaOH solution concentration is fixed at 10 mol/L. The results shown in Figure 8 indicate that the increased slag replacement rate can significantly improve the compressive strength. For the specimens with a fixed concentration of 10 mol/L, when the slag rate changes from 20% to 60%, the increased range of the peak compressive strength is over 100%. The explanation for this is that the increase in the slag content increases the ratio of Si/Al in the mixed materials. This indicates that the proportion of Si present in the mix determines the relative amount of AlO_4_ and SiO_4_, which results in the formation of the aluminosilicate-hydrate (C-A-S-H) gel and contributes to the improvement in compressive strength. For the specimens with a slag rate that is larger than 20%, the SSGRC exhibits a better performance. For the specimens with a slag ratio of 40% and 60%, the peak stress of the specimens with sea sand is 22.6% and 10.8% higher than those with river sand, respectively. On the one hand, the higher value for the specimen with sea sand is attributed to the chemical substances such as free sodium ions, calcium ions, magnesium ions, and potassium ions from the sea sand, which help accelerate the hydration process of C_3_S and C_2_S. Moreover, some of these hydration products react to form new hydration products such as 3CaO·Al_2_O_3_·CaCl_2_·10H_2_O, which contribute to the strength. On the other hand, these hydration products have microparticle threads inside the older mortar and interface transition zone, resulting in a decrease in voids and pores within the RCAs.

**Figure 9:**
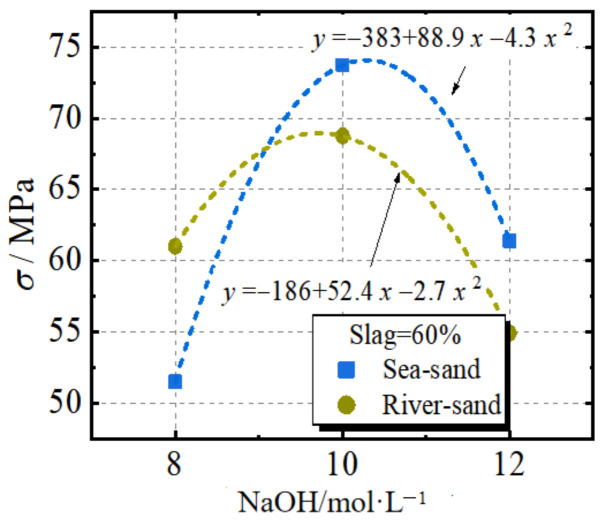
The effect of NaOH solution concentration on the compressive strength.

Figure 9 depicts the effect of the NaOH solution molarity on the compressive strength. To investigate the effect of NaOH molarity, all of the specimens had a slag replacement rate of 60%. It can be seen from Figure 9 that as the molarity of the NaOH solution increases from 8 M to 10 M, the peak stress increases by 41.3% and 11.7% for the specimens with sea sand and river sand, respectively. However, when the molarity increases from 10 M to 12 M, the peak stress decreases. Hence, the 10 M NaOH solution is regarded as the optimum molarity. This can be explained by the geopolymer product depending on the hydroxide ion (OH) that is mainly derived from NaOH. More hydroxide ions improve the strength of the product. However, the formation of aluminosilicate gel during geopolymerization can cause precipitate at an early stage and result in an excessively alkaline environment. Hence, a high NaOH molarity decreases the strength. With the increase in the molarity of the NaOH solution, the development of compressive strength first increased and then declined. An excessively high NaOH solution molarity leads to a high alkaline environment, which hinders Ca(OH)_2_ dissolution. Due to the insufficient amount of Ca^2+^, the predominate hydration product is converted from C-A-S-H to N-A-S-H. According to this trend, we can assume that the compressive stress increases based on the form of the quadratic parabola function with the increasing molarity of the NaOH solution. Based on the experimental results, predictions using Equations (3) and (4) were proposed to obtain a reasonable NaOH molarity depending on the target strength. In these equations, *n* represents the molarity of the NaOH solution.

(3) $$\sigma =-383+88.9n-4.3n^{2}$$

(4) $$\sigma =-186+52.4n-2.7n^{2}$$

## 5. Numerical Compressive Stress–Strain Relationship

At present, a large number of constitutive models have been proposed for ordinary concrete [34,35,36]. However, there are few studies that give a specific numerical expression for geopolymer recycled concrete. The model proposed by Guo [34] is commonly used for ordinary concrete and recycled aggregate concrete. In this paper, taking into account the characteristics of geopolymer recycled concrete, a numerical model for SSGRC is proposed by extending the model from Guo’s study to be applied to this concrete.

In order to facilitate the establishment of the constitutive equations of geopolymer recycled concrete, the stress–strain curve in Figure 6 is normalized by processing it according to Equation (5).

(5) $$x=\varepsilon /\varepsilon_{p}\;\;,\;\;\;\;y=\sigma /\sigma_{p}\;\;$$

where *x* and y are the normalized stress and strain; *σ*_p_ represents the peak stress; and *ε*_p_ represents the strain corresponding to the peak stress.

The normalized expression for the stress–strain curve of geopolymer recycled concrete can be approximated using Equation (6).

(6a,b) $$y=\left\{\begin{array}{c} Ax+\left(3-2A\right)x^{2}+\left(A-2\right)x^{3}\;\;,\;\;\;\;x\leq 1\\ \frac{x}{B{\left(x-1\right)}^{2}+x}\;\;\;\;,\;\;\;\;\;\;\;\;x\geq 1 \end{array}\right.$$

The results of the regression analysis are shown in Table 5. In Equation (6a), parameter *A* represents the initial slope of the stress–strain curve in the ascending segment. Based on the experimental data in this paper, the average value of parameter A obtained through regression analysis using the modified model is 0.622.

In Equation (6b), parameter *B* is the area in the descending segment of the stress–strain curve. It represents the ductility of the concrete. Based on the experimental data, and considering the effect of curing age t, parameter *B* can be described using Equation (7).

(7) $$B=\frac{t}{0.41+0.21t}$$

where *t* represents the curing age.

The modified compression constitutive model is obtained as follows:

(8) $$y=\left\{\begin{array}{c} 0.622x+1.756x^{2}-1.378x^{3}\;\;,\;\;\;\;\;\;\;\;\;\;x\leq 1\\ \frac{x}{\frac{t}{0.41}+0.21t{\left(x-1\right)}^{2}+x}\;\;\;,\;\;\;\;\;\;\;\;x>1,\;\;1d\leq t\leq 28d \end{array}\right.$$

**Figure 10:**
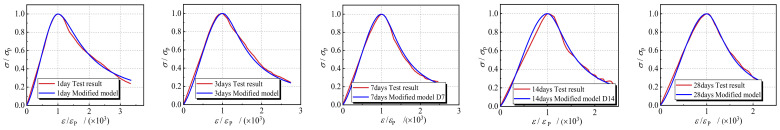
The comparison between modified model curves and test curves.

The comparison between the stress–strain curves of the modified model and the test results is shown in Figure 10. It can be found that the calculated curves of the modified model are basically consistent with the experimental ones. Hence, the modified model can be considered applicable for theoretical analysis and practical engineering design.

## 6. Conclusions

In this paper, 42 specimens were tested in the axial compressive loading experiment to investigate the effects of the fine aggregate type, slag replacement rate, NaOH molarity, and curing age on the mechanical properties of geopolymer-based recycled concrete. The detailed conclusions are as follows:

1. As the curing age increases, the color of the failure surface changes from dark to light. Additionally, the increase in the NaOH molarity can effectively improve the bonding performance, resulting in cracks in the RCAs instead of interface debonding.
2. According to the SEM analysis, it is shown that the geopolymerization product can fill the voids of the porous aggregate particles and provide a favorable bonding performance between the paste and the RCAs.
3. The stress–strain curve of geopolymer concrete is similar to the compressive stress curve of ordinary concrete. The compressive strength of geopolymer concrete at 1 and 7 days can reach 56% and 86% of the compressive strength at 28 days. The geopolymer concrete exhibits a rapid increase in compressive strength. The relationship between the strength and curing age for the geopolymer concrete with sea sand and river sand is proposed.
4. For the specimens with a fixed NaOH concentration of 10 mol/L, with the increase in the slag replacement rates from 20% to 60%, the improvement in the compressive strength is achieved and is over 100%. Meanwhile, the increase in the elastic modulus is also observed. Replacing river sand with sea sand is feasible in terms of compressive strength.
5. For both the sea sand and river sand, the optimum molarity value of the NaOH solution is around 10 M. Compared with the molarities of 8 M and 12 M, the compressive strength when the molarity is 10 M increases by 53.7% and 17.6%, respectively, for concrete with sea sand. For concrete with river sand, the increase in compressive strength when the molarity is 10 M is 25.9% and 12.6%. The NaOH solution can accelerate the geopolymerization process. An excessive alkaline environment causes the precipitation of the geopolymerization product. The relationship between the molarity of the NaOH solution and the compressive strength is established.
6. Based on the experimental data, a modified compressive constitutive model for geopolymer recycled concrete is proposed that considers the curing age. The calculated curves are consistent with the test results.

## Figures and Tables

**Table 1:** Chemical composition of fly ash and slag (%).

	SiO_2_	Al_2_O_3_	CaO	Fe_2_O_3_	MgO	SO_3_	TiO_2_
Fly ash	49.32	31.20	3.23	8.23	1.03	0.85	0.97
Slag	32.80	15.36	37.12	0.74	8.52	-	1.95

**Table 2:** Basic physical properties of the coarse aggregate.

	Apparent Density (kg·m^−3)^	Bulk Density (kg·m^−3)^	Water Absorption Rate (%)	Crush Index (%)	Attached Mortar (%)
RCA	2600.0	1321.4	5.4	25.6	32.3

**Table 3:** The design parameters of the mechanical properties test.

Specimen Label	Curing Age (Days)	NaOH/ (mol/L)	Slag (%)	Sand Types	Loading Scheme	Number
D1-10-60-S	1	10	60	Sea sand	compression	3
D3-10-60-S	3	10	60	Sea sand	compression	3
D7-10-60-S	7	10	60	Sea sand	compression	3
D14-10-60-S	14	10	60	Sea sand	compression	3
D28-8-60-S/R	28	8	60	Sea/River sand	compression	6
D28-10-20-S/R	28	10	20	Sea/River sand	compression	6
D28-10-40-S/R	28	10	40	Sea/River sand	compression	6
D28-10-60-S/R	28	10	60	Sea/River sand	compression	6
D28-12-60-S/R	28	12	60	Sea/River sand	compression	6

Notes: D = curing days; S = sea sand; R = river sand.

**Table 4:** Compressive strength of all mixes at different curing times.

Sand Type	Molarity of NaOH (mol/L)	Slag Replacement Rate (%)	Compressive Strength (MPa)				
			1 Day	3 Days	7 Days	14 Days	28 Days
Sea sand	10	20	-	-	-	-	23.73
		40	-	-	-	-	51.48
		60	34.4	50.0	63.3	59.3	73.1
	8	60	-	-	-	-	51.0
	12	60	-	-	-	-	65.5
River sand	10	20	-	-	-	-	27.0
		40	-	-	-	-	37.9
		60	38.1	48.7	58.7	68.9	68.3
	8	60	-	-	-	-	62.6
	12	60	-	-	-	-	60.4

**Table 5:** Summary of parameters of modified mode.

Specimen	Curing Age (Days)	Ascending Section				Descending Section		
		*A*	R^2^	Average	Variance	*B*	R^2^	Formula
D1-10-60-S	1	0.49	0.999	0.622	0.028	1.65	0.987	$B=\frac{t}{0.41+0.21t}$
D3-10-60-S	3	0.81	0.999			2.64	0.996	
D7-10-60-S	7	0.66	0.992			4.04	0.995	
D14-10-60-S	14	0.41	0.961			4.11	0.993	
D28-10-60-S	28	0.74	0.996			4.3	0.997	

## Data Availability

Data are contained within the article.
